# Polymorphonuclear Leukocyte Transendothelial Migration Proceeds at Blood-Brain Barrier in Neonatal Meningitis

**DOI:** 10.3389/fmicb.2020.00969

**Published:** 2020-05-26

**Authors:** Zhuo Niu, Yu-Hua Chen, Ke Zhang

**Affiliations:** ^1^Department of Developmental Cell Biology, Key Laboratory of Cell Biology, Ministry of Public Health, Key Laboratory of Medical Cell Biology, Ministry of Education, China Medical University, Shenyang, China; ^2^Department of Surgical Oncology and General Surgery, Key Laboratory of Precision Diagnosis and Treatment of Gastrointestinal Tumors, Ministry of Education, The First Affiliated Hospital of China Medical University, Shenyang, China

**Keywords:** polymorphonuclear leukocyte, transendothelial migration, blood-brain barrier, brain microvascular endothelium, neonatal meningitis, bacteria, host

## Abstract

Neonatal bacterial meningitis remains a life-threatening and causative sequelae disease in newborns, despite the effective usage of antibiotics and improved critical medical care. Polymorphonuclear leukocyte (PMN) transendothelial migration across the blood-brain barrier, one of the three hallmarks of bacterial meningitis, now is considered as a “double-edge sword”. When participating in host immune system defending against virulent pathogens, it results in tissue inflammation and following severe damage of central nervous system at the same time, which contributes to a disastrous consequence. Recently, several researches have focused on this multi-step process and the mechanism of how the virulent factors of different pathogens influence PMN migration. The great progression they made has enlightened a new research hotspot and a novel therapeutic strategy. This mini review outlines the determinants and progression of PMN transmigration in neonatal meningitis caused by different predominant pathogens.

## Introduction

Neonatal bacterial meningitis is regarded as a lethal and causative sequelae disease among newborns. Despite the process of the rapid diagnosis of pathogens and availability of effective bactericidal antibiotics, neonatal meningitis maintains a high neonatal mortality and morbidity worldwide (Huang et al., 2000; Lawrence et al., 2015). The epidemiology for neonatal bacterial meningitis varies from country to country (Zaidi et al., 2009; Khalessi and Afsharkhas, 2014). In general, in developed countries, the incidence is estimated at 0.3 per 1000 live births and mortality ranges 10–15%. Meanwhile, the major pathogens are group B Streptococcus (GBS), *Escherichia coli* (*E. coli*), and *Listeria monocytogenes* (*L. monocytogenes*). However, the incidence remains much higher at 0.8–6.1 per 1000 live births and 40–58% of neonates dies from it in developing countries where the important microorganisms are GBS, other Gram negatives (excluding *E. coli*), *L. monocytogenes* and Gram-positive organisms, respectively (Zaidi et al., 2009; Furyk et al., 2011; Khalessi and Afsharkhas, 2014; Lawrence et al., 2015). The successful meningitic pathogens must enter the peripheral blood to form the bacteremia and cross the blood-brain barrier (BBB) which anatomically have a characteristic tight junction of brain microvascular endothelial cells (BMEC) (Bowman et al., 1983; Rubin and Staddon, 1999; Huang et al., 2000). Different bacteria use different mechanisms to cross the BBB. In the past several decades, microbiologists focused more on identified virulence factors in bacteria that taking part in their traversal of the BBB in bacterial meningitis. However, pathogens crossing the BBB is only the primitive step during the progression of bacterial meningitis. In the subsequent process of the bacterial meningitis following the entry of bacteria into the cerebrospinal fluid, polymorphonuclear leukocyte (PMN) transendothelial migration across the BBB is another substantial feature of bacterial meningitis. The penetration of PMN across BBB is regard as a “double-edge sword”. On one hand, PMN can help the host defense fight against pathogens; oppositely, PMN may also cause significant tissue damage to the central nervous system (CNS), leading to the serious neurologic sequelae (Flier et al., 2003; Wang et al., 2016). In bacterial meningitis, PMN transendothelial migration is a multi-step process involving pathogen, neutrophil, as well as BMEC. This mini review outlines the determinants and progression of PMN transmigration in neonatal meningitis caused by different predominant pathogens.

## Bacterial Meningitis Caused by *Escherichia Coli*

*Escherichia coli* is the most common Gram-negative bacterium causing neonatal meningitis, which is also the main pathogen in developing countries (Khalessi and Afsharkhas, 2014). The bacteria with K1 capsule is the dominant (∼80%) serotype in *E. coli* meningitis (Huang et al., 2000). IbeA (invasion brain endothelial protein A) was firstly validated as a vital determinant of *E. coli* K1 to promote bacterial penetration across the BBB (Huang et al., 1995). IbeA also played an important role in facilitating PMN transmigration. Not only ibeA^+^ *E. coli* K1, purified ibeA protein can induce PMN transmigration independently (Che et al., 2011; Chi et al., 2012). IbeA, presenting on the outer membrane of *E. coli* K1, can interact with its primary receptor vimentin together with its co-receptor PTB-associated splicing factor (PSF) on BMEC to activate NF-κB (nuclear factor kappa-light-chain-enhancer of activated B cells) signaling, which consequently accelerates the recruitment the PMN to BMEC (Chi et al., 2012; Huang et al., 2016). Meantime, ibeA^+^ *E. coli* K1 and purified ibeA protein triggered the upregulation of adhesion molecules containing intercellular adhesion molecule-1 (ICAM-1) and CD44, which were involved in the PMN movement across the BMEC monolayer through the enhanced adhesion of PMN to BMEC (Che et al., 2011). FimH (type 1 fimbrial tip adhesin), expressed on *E. coli* K1, made the bacteria have a mannose-sensitive binding ability, which is another important determinant participating not only in bacterial adhesion and invasion, but also in PMN transmigration (Liu et al., 2019). The fimH knock-out mutant in *E. coli* K1 presented inefficiency in promoting PMN transmigration compared with wild-type *E. coli* K1 (Chi et al., 2011). FimH in *E. coli* K1 can mediate PMN transmigration across BMEC through binding to the protein complex composed of CD48 (the receptor of fimH) and alpha 7 nicotinic acetylcholine receptor (α7 nAChR) on the lipid raft of BMEC (Chi et al., 2011). Previous study showed that α7 nAChR plays a critical role in modulation of pathogen invasion and PMN recruitment in *E. coli* meningitis as an independent factor. In addition, fimH interacted with CD48/α7 nAChR complex to active Ca^2+^ signaling and induce cofilin dephosphorylation, which may be the probable molecular mechanism for PMN transmigration mediated by fimH (Chi et al., 2011; Liu et al., 2019).

Recently, we found *E. coli* K1 virulence factor cglD promote PMN transendothelial migration (Zhang et al., 2009, 2019). *CglD* and *ibeA* genes are located at the same pathogenicity island named GimA (genetic island of meningitic *E. coli* containing *ibeA*) (Huang et al., 2001). The former has a 1083-bp open reading frame (ORF) and the product of *cglD* gene expression has the activity of glycerol dehydrogenase (Zhang et al., 2009). Different from other virulence factors, cglD did not participate in the invasion of the BMEC (Zhang et al., 2009). Meanwhile, cglD in *E. coli* K1 may bear a part in the activation of the NF-κB signaling pathway in BMEC. This results in the release of some inflammation-related cytokines, including CXCL1 (chemokine (C-X-C motif) ligand 1), CXCL6 and CXCL8, which enhanced the attraction of PMN to BMEC. Meantime, with the increase of E-selectin expression in BMEC, the adhesion of PMN to BMEC are strengthened, which ultimately promotes transendothelial migration across the BBB into the brain (Zhang et al., 2019). Although there is a crosstalk between *E. coli* and BMEC, the molecular and cellular mechanisms for PMN transmigration remain to be defined. Further detailed study is needed to discover the novel therapeutic targets which can be modulated to make the PMN playing more positive roles in bacterial meningitis progression.

## Bacterial Meningitis Caused by Group B Streptococcus

Group B Streptococcus is the most frequent Gram-positive bacterium and also a leading cause of meningitis in newborn infants in developed country such as United States (Khalessi and Afsharkhas, 2014). GBS capsular serotypes III strain is commonly associated with bacteremia and GBS meningitis develops as a consequence when pathogens move across the BBB (Nizet et al., 1997). BMEC challenged with GBS produces some functional genes expression products including IL-8 (interleukin 8), Gro α (growth-related gene product α)/CXCL1, Gro β/CXCL2, IL-6, GM-CSF (granulocyte-macrophage colony-stimulating factor), myeloid cell leukemia sequence-1 (Mcl-1), and ICAM-1 (Doran et al., 2003). Among them, IL-8, Gro α, and Gro β are responsible for the recruitment of PMN; GM-CSF is contributed to stimulating PMN produced from bone marrow; ICAM-1 and Mcl-1 are responsible for the adhesion of PMN to BMEC and prevention of PMN apoptosis, respectively (Doran et al., 2003). These specific expressions of genes are mainly mediated by β-hemolysin/cytolysin toxin (β-h/c) of GBS (Doran et al., 2003). Deletion of β-h/c results in a significantly reduction in expression of these genes when the BMEC infected with β-h/c deletion mutant strain, consequently causes decreased PMN transmigration (Doran et al., 2003). Therefore, β-h/c plays an important role in neutrophil across the BMEC, furthermore, Doran et al. (2003) showed that capsular polysaccharide in GBS is not essential for the recruitment of PMN transmigration. Pili, cell surface appendage, was recently discovered in GBS, which is contributed to the adhesion of GBS to brain microvascular endothelium (Maisey et al., 2007). The gene *pilA* was identified to participate in assembling the pili and the expression of gene *pilA* has a positive effect on the adhesion of GBS (Nobbs et al., 2008). With further study, PilA–GST proteins can induce a significant release of IL-8 by BMEC; accordingly, PilA-deficient mutant caused a downregulation of IL-8 in BMEC, which results in a reduced PMN recruitment to BMEC (Banerjee et al., 2011). PilA has the capacity to interact with collagen, which typically binds to α2β1 integrins to initiate the activation of FAK (focal adhesion kinase) and subsequent PI3K (phosphoinositide 3-kinases) and MEK1/2 (MAPK/ERK kinases)-ERK1/2 (extracellular signal regulated kinase) signaling pathway in BMEC. These events lead to the release of IL-8 and neutrophil recruitment by BMEC and ultimately enhance the PMN transmigration (Banerjee et al., 2011). Lately, surface antigen I/II protein BspC was verified in GBS, which was studied as a multifunctional adhesins in other Streptococci. Beside the traditional adherent and invasive function, BspC also takes part in the PMN transmigration through stimulating the activation of NF-κB signaling pathway and expression of IL-8 and CXCL1. During this process, cytoskeletal component vimentin in BMEC made a great contribution through interacting with BspC in BMEC (Deng et al., 2019). It is worth noting that vimentin expressed by BMEC has a cross talk with a variety of bacteria, which indicated that vimentin is a potential target to regulate the PMN transendothelial migration (Tim and Holger, 2016).

## Bacterial Meningitis Caused by Other Bacteria

In addition to *E. coli* and GBS, *L. monocytogenes* is the third common reported bacteria which can cause the neonatal meningitis (Disson and Lecuit, 2012; Khalessi and Afsharkhas, 2014). *L. monocytogenes* is a Gram-positive bacterium widely spread in soil, animals and human. Although reported as a rare type of meningitis, it is always life-threatening, because *L. monocytogenes* is nearly tenfold more efficient in invading the CNS than other Gram-positive bacteria, including GBS (Schuchat et al., 1997). At present, there is no exact study on PMN transendothelial migration in *L. monocytogenes* meningitis. But *L. monocytogenes* can stimulate the expression of P-selectin, E-selectin, ICAM-1 and VCAM-1 (vascular cell adhesion molecule 1), as well as IL-8 and MCP-1 (monocyte chemoattractant protein 1) both in BMEC and brain microvessels through activating NF-κB signaling pathway, which enhance the adhesion of neutrophil to BMEC (Krüll et al., 1997; Wilson and Drevets, 1998; Kayal et al., 1999, 2002). During this process, the pore-forming toxin Listeriolysin O (LLO) in *L. monocytogenes* makes a great contribution in triggering PMN adhesion to BMEC by facilitating and enhancing the expression of these functional proteins (Al Obaidi and Desa, 2018). In *Neisseria meningitidis (N. meningitidis)* meningitis, the bacteria boost firm adhesion to BMEC, and then PMN transendothelial migration is inhibited ultimately, which is different from other neuroinvasive bacteria (Doulet et al., 2006). Doulet et al. (2006) explained that an “endothelial docking structures” consisted of actin-rich membrane protrusion caused by the adhesion of PMN on BMEC is required for the PMN transmigration. While BMEC are infected with *N. meningitidis*, BMEC effectively recruit ezrin and moesin (known as ERM), and ezrin binding adhesion molecules, such as ICAM-1, ICAM-2, VCAM-1, and CD44. The segregation of ERM and these adhesion molecules caused by *N. meningitidis* results in the abolishment of “endothelial docking structures,” which lead to the failure of PMN transmigration (Doulet et al., 2006).

## Discussion

Bacterial meningitis usually displays triad hallmark features: pathogen penetration, NF-κB activation and leukocyte transmigration. Although antibiotics and critical medical care have improved, the prognosis is still unsatisfying. Together with the severe “side effect” of CNS inflammation following the pathogens across BBB, the PMN transendothelial migration has been brought to our attention. Several researches about different determinant pathogens have made and the molecular and cellular mechanisms have been revealed. Among these, *E. coli* and GBS, reported as the leading pathogens, are well studied, respectively. But the detailed mechanism should be defined in the future. For example, the cglD in *E. coli* K1 is a cytoplasmic protein. The mechanism of how it activates NF-κB signaling pathway in BMEC is needed further investigation. The influence of other common pathogens on PMN migration also is needed to be explored.

**FIGURE 1:**
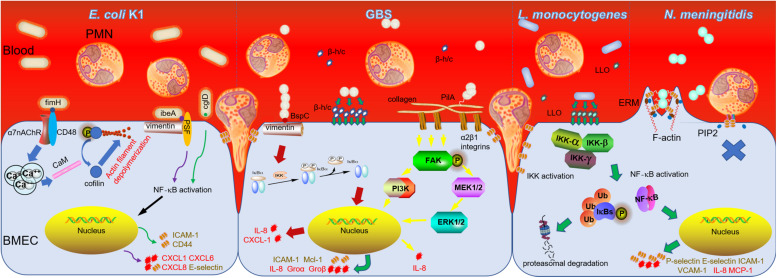
PMN transmigration in bacterial meningitis caused by *E. coli* K1, GBS, *L. monocytogenes*, and *N. meningitidis*. The secreted toxins and other virulent factors presenting either on or in the pathogens mainly interact with receptors of BMEC which transduce signals to nucleus and induce expression of numerous chemoattractive cytokines and adhesion molecules to promote PMN migration across BBB ultimately, while *N. meningitidis* adheres to BMEC to form a “tight” junction which prevents PMN transendothelial migration subsequently.

Overall, PMN transendothelial migration occurring in neonatal meningitis is driven by interactions between meningitic pathogens and brain microvascular endothelium. The molecular and cellular mechanisms about these interactions in neonatal meningitis have been revealed by basic medical research and crucial bacterial pathogenic determinants and host factors have been explored (Table 1 and Figure 1). In future, discovering whether they share a common strategy to influence neutrophil transmigration, such as the vimentin, will bring us a novel therapeutic strategy. Furthermore, along with more and more attempt to regulate PMN transmigration, prevention of bacterial meningitis progression will make an improvement.

**TABLE 1:** Summary of pathogenic features on PMN transmigration in bacterial meningitis.

Pathogen	Virulent factor	Host cell receptor	Signaling pathway	Downstream factor	References
*E. coli* K1	ibeA	vimentin, PSF	NF-κB	ICAM-1, CD44	Huang et al., 1995; Che et al., 2011; Chi et al., 2012; Huang et al., 2016
	fimH	CD48, α7 nAChR	Ca^2+^ signaling	cofilin dephosphorylation	Chi et al., 2011; Liu et al., 2019
	cglD	unknown	NF-κB	CXCL1, CXCL6, CXCL8, E-selectin	Zhang et al., 2009; Zhang et al., 2019
GBS	β-h/c	unknown	unknown	IL-8, Gro α, Gro β, GM-CSF, ICAM-1, Mcl-1	Doran et al., 2003
	PilA	collagen, α2β1 integrins	FAK, PI3K, MEK1/2, ERK1/2	IL-8	Banerjee et al., 2011
	BspC	vimentin	NF-κB	IL-8, CXCL1	Deng et al., 2019
*L. monocytogenes*	LLO	unknown	NF-κB	P-selectin, E-selectin, ICAM-1, VCAM-1, IL-8, MCP-1	Krüll et al., 1997; Wilson and Drevets, 1998; Kayal et al., 1999; Kayal et al., 2002; Al Obaidi and Desa, 2018
*N. meningitidis*	type IV pili	CD44, ERM, PIP2, ICAM-1, F-actin			Doulet et al., 2006

## Author Contributions

KZ conceived and designed the mini review. ZN and KZ wrote the manuscript. ZN, Y-HC, and KZ corrected the manuscript.

## Conflict of Interest

The authors declare that the research was conducted in the absence of any commercial or financial relationships that could be construed as a potential conflict of interest.
